# SirT1 and rRNA in the nucleolus: regulating the regulator

**DOI:** 10.18632/oncoscience.15

**Published:** 2014-03-15

**Authors:** Leixiang Yang, Jiandong Chen

**Affiliations:** Molecular Oncology Department, Moffitt Cancer Center, Tampa, FL

The synthesis of ribosomal RNA (rRNA) is tightly coupled to nutrient conditions and growth factors. Murayama et al. described a novel nucleolar silencing complex eNoSC involved in rRNA transcription regulation in response to glucose deprivation [1]. This complex contains SirT1, SUV39H1 and the nucleolar protein nucleomethylin (NML). Upon glucose starvation, NML inhibits rRNA synthesis by recruiting SirT1 and SUV39H1 to rDNA promoter and spreading heterochromatin marks across the rDNA repeat.

Unlike yeast Sir2, which localizes to the nucleolus and telomeres, mammalian SirT1 is predominantly located in the nucleoplasm. Therefore, SirT1 function in the nucleolus may require recruitment by the nucleolar NML. In a recent study, we investigated the dynamic regulation of eNoSC assembly by nutrient conditions and found that the interaction of SirT1 and NML was regulated by rRNA [2]. A disordered region adjacent to the methyltransferase domain of NML was found to bind RNA in a sequence- independent manner *in vitro*. The RNA and SirT1 binding sites on NML overlap partially. Thus, RNA and SirT1 compete for interacting with NML. The NML complex purified from cells contains predominantly ribosomal rRNA (5S, 5.8S and 28S), presumably due to its nucleolar localization. The interaction of NML with rRNA *in vivo* was inhibited by glucose deprivation, which is known to down-regulate pre-rRNA transcription through the AMPK-mTOR-RNA Pol I signaling pathway. Therefore, glucose deprivation strongly stimulates NML-SirT1 binding, resulting in the recruitment of a minor fraction of SirT1 to the nucleolus. Assembly of eNoSC contributes to the down regulation of rRNA transcription in the absence of glucose. In fact, various stresses that down regulate rRNA transcription, such as DNA damage, inhibition of mTOR using rapamycin, or direct inhibition of RNA Pol I using actinomycin D or CX5461, all promote SirT1-NML binding.

**Figure 1:**
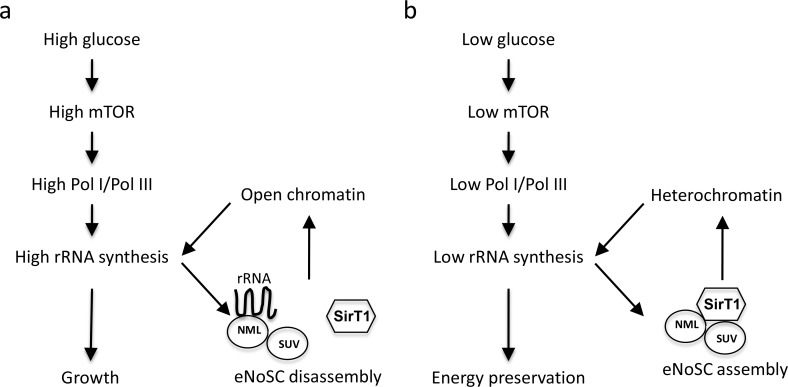
A model of nutrient-dependent regulation of NML-SirT1 interaction (a) During nutrient-rich growth, mTOR stimulates RNA Pol I and Pol III synthesis of rRNA. The nascent rRNA in turn binds to NML and inhibits eNoSC assembly on rDNA, providing a positive feedback to amplify mTOR signaling. (b) During starvation, mTOR inactivation reduces Pol I and Pol III activity. Reduced nascent rRNA level enables NML to bind and recruit SirT1 to the nucleolus, promoting heterochromatin formation and silencing of the rDNA.

These findings led to a positive feedback model that coordinates SirT1 function in the nucleolus with nutrient signaling: Under nutrient-rich condition, mTOR and other factors stimulate RNA Pol I and Pol III synthesis of rRNA. Nascent rRNA is processed and assembled into pre-ribosomal particles at the nucleolus. The abundant nascent rRNA also interacts with NML and prevents the recruitment of SirT1. This mechanism provides a positive feedback loop to nutrient signaling by protecting the active nucleolar rDNA from repression by the NML- SirT1 complex (Figure 1a). During starvation, mTOR inactivation reduces Pol I and Pol III activity. Reduced level of nascent rRNA enables NML-SirT1 complex formation in the nucleolus, promoting heterochromatin formation and silencing of the rDNA (Figure 1b). Therefore, NML provides positive feedback on mTOR regulation of rRNA synthesis by sensing the level of the nascent rRNA output.

SirT1 is well known for its regulation by both the substrate and the reaction products. NAD+ is required for SirT1-mediated deacetylation reaction, whereas the deacetylation byproduct nicotinamide is an inhibitor of SirT1 activity [3]. Assembly of the SIR complex in yeast is inhibited by another product of deacetylation, O-acetyl- ADP-ribose [4]. Our study identified a novel mechanism of SirT1 regulation that involves the RNA product of transcription. Furthermore, the results suggest that the RNA-mediated regulation may not require sequence specificity, but is determined primarily by spatial proximity. The co-localization of NML and nascent rRNA in the nucleolus ensures that rRNA level has a specific impact on NML-SirT1 interaction. Since nascent rRNA is rapidly assembled into pre-ribosomal particles at the nucleolus, it is possible that the levels of ribosomal proteins also regulate NML-SirT1 binding to provide feedback to rRNA transcription.

The new findings on NML provides another example of sequence non-specific RNA binding similar to several heterochromatin proteins such as HP1^swi6^ and Chp1 from fission yeast [5, 6]. HP1^swi6^ binds RNA through a positively charged flexible region, whereas Chp1 binds RNA through a flexible sequence in its chromodomain. RNA binding has diverse functional effects on these proteins. RNA competes with the HP1^swi6^ chromodomain for binding to H3K9me, stimulates Chp1 chromodomain binding to H3K9me, and inhibits NML binding to SirT1. It is possible that sequence non-specific RNA binding through unstructured region is an important feature of heterochromatin proteins, which allow them to be regulated at many chromosomal loci by locally produced coding and non-coding RNA.

In addition to regulation of NML-SirT1 binding, it is possible that RNA may regulate other biochemical activities of NML. NML has a methyltransferase- like domain at the C-terminus; but its substrate is still unknown. Murayama et al. has excluded histone proteins as the target of NML [1]. NML is the presumptive human homolog of the yeast ribosomal RNA processing protein 8 (Rrp8) that was previously shown to be involved in rRNA cleavage and 40S biogenesis in yeast [7]. Recently, it has been reported that Rrp8 is able to modify m1A 645 base of 25S rRNA in yeast [8]. Therefore, NML may be involved in the methylation of nucleic acids rather than proteins. Misregulation of nucleolar transcription is a common feature of tumor cells. It has been demonstrated that depletion of nucleolar repressor complex NoRC subunit TIP5 promotes transformation. Given its role as a nutrient-responsive regulator of rDNA transcription, NML dysfunction may contribute to tumor development. These questions should be addressed in future studies.
